# Reproducibility of sequential ambulatory blood pressure and pulse wave velocity measurements in normotensive and hypertensive individuals

**DOI:** 10.1097/HJH.0000000000003290

**Published:** 2022-09-27

**Authors:** Louise Keehn, Wendy L. Hall, Sarah E. Berry, Thomas A.B. Sanders, Phil Chowienczyk, Christopher N. Floyd

**Affiliations:** a King's College London British Heart Foundation Centre, Department of Clinical Pharmacology, St Thomas’ Hospital; b Department of Nutritional Sciences, Franklin-Wilkins Building, King's College London, London, UK

**Keywords:** ambulatory, ambulatory blood pressure monitoring, blood pressure, hypertension, reproducibility

## Abstract

Errors in blood pressure (BP) measurement account for a large proportion of misclassified hypertension diagnoses. Ambulatory blood pressure monitoring (ABPM) is often considered to be the gold standard for measurement of BP, but uncertainty remains regarding the degree of measurement error. The aim of this study was to determine reproducibility of sequential ABPM in a population of normotensive and well controlled hypertensive individuals.

**Methods:**

Individual participant data from three randomized controlled trials, which had recorded ABPM and carotid-femoral pulse wave velocity (PWV) at least twice were combined (*n* = 501). We calculated within-individual variability of daytime and night-time BP and compared the variability between normotensive (*n* = 324) and hypertensive (*n* = 177) individuals. As a secondary analysis, variability of PWV measurements was also calculated, and multivariable linear regression was used to assess characteristics associated with blood pressure variability (BPV).

**Results:**

Within-individual coefficient of variation (CoV) for systolic BP was 5.4% (day) and 7.0% (night). Equivalent values for diastolic BP were 6.1% and 8.4%, respectively. No statistically significant difference in CoV was demonstrated between measurements for normotensive and hypertensive individuals. Within-individual CoV for PWV exceeded that of BP measurements (10.7%). BPV was associated with mean pressures, and BMI for night-time measurements. PWV was not independently associated with BPV.

**Conclusion:**

The variability of single ABPM measurements will still yield considerable uncertainty regarding true average pressures, potentially resulting in misclassification of hypertensive status and incorrect treatment regimes. Repeated ABPM may be necessary to refine antihypertensive therapy.

## Graphical abstract

**Figure:**
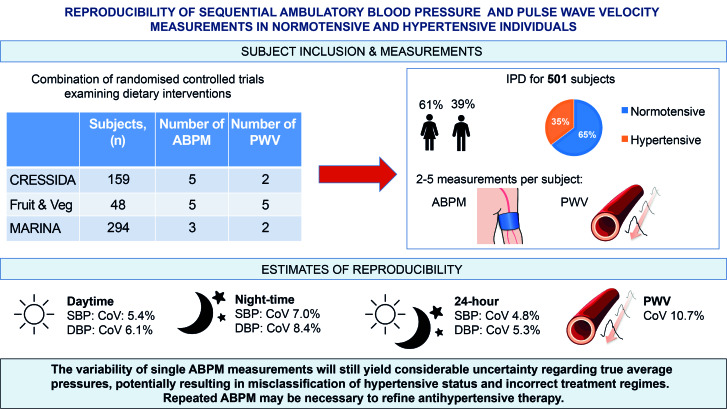

## INTRODUCTION

Ambulatory blood pressure measurement (ABPM) has been shown to be the most cost-effective option to confirm a diagnosis of hypertension [1]. The reproducibility of average blood pressure (BP) taken by 24-h ABPM has been previously shown to be superior to the reproducibility of clinic BP [2–4]. However, the majority of studies examining ABPM reproducibility have been performed with time intervals of 12 weeks or less, or in individuals with long-term hypertension or a history of cardiovascular disease.

There are fewer studies comparing techniques for long-term monitoring and clinic BP remains a first-line tool despite well known risks from white-coat or masked hypertension [5–7]. Long-term monitoring using ABPM could facilitate improved BP control, but the degree of variability between sequential ABPM in normotensive and stable hypertensive individuals otherwise free from overt cardiovascular disease is poorly characterized.

A retrospective analysis of individual patient data (IPD) from randomized controlled trial (RCTs) provides an opportunity to investigate ABPM measurement variability. Here, we present analyses of measurement variability from three studies, which investigated possible benefits of dietary modification on ambulatory BP and arterial stiffness in individuals who were normotensive or with well controlled hypertension [8–10]. The concurrent measurement of pulse wave velocity (PWV) in these individuals presented an ideal opportunity to directly compare the reproducibility of PWV against that of ABPM, as superior reproducibility may support the alternate use of PWV as a long-term monitoring tool for cardiovascular health.

Even in a healthy population, reproducibility of BP measurements will be affected by blood pressure variability (BPV). Some degree of BPV is a normative property, but a high variability has been shown to be associated with an increased risk of cardiovascular outcomes, independent of the mean systolic pressure [11–13]. Determinants of increased BPV may include general cardiovascular risk factors such as increasing age, arterial stiffness and adverse lipid profiles [14,15] amongst others. Elucidation of factors associated with BPV in this cohort may provide clues to modifiable risk factors for high BPV in cohorts at a higher risk of cardiovascular morbidities.

Therefore, the primary aim of the present study was to calculate reproducibility associated with sequential ABPM in this relatively healthy population of normotensive and well controlled hypertensive individuals. Secondary aims were to estimate BPV and its potential determinants and to compare the reproducibility of arterial stiffness to that of ABPM for evaluation of its use as a surrogate technique for long-term measurement of vascular health.

## MATERIALS AND METHODS

### Individuals and inclusion criteria

We analysed IPD from three RCTs investigating the impact of dietary modifications on cardiovascular outcomes. Firstly, the Fruit & Veg study (ISRCTN50011192) tested whether a potassium-rich diet was beneficial for treatment-naive prehypertensive individuals (*n* = 48) [8]. Secondly, the MARINA study (ISRCTN666664610, *n* = 312) examined if increasing intake of long-chain n-3 polyunsaturated fatty acids favourably affected endothelial function and arterial stiffness [9]. Finally, the CRESSIDA study (ISRCTN9282106, *n* = 162) considered how following UK dietary guidelines, instead of a traditional British diet, might affect vascular function [10]. All study and trial procedures were performed at Guy's and St. Thomas’ NHS Foundation Trust. Each study was approved by a local research ethics committee.

**FIGURE 1:**
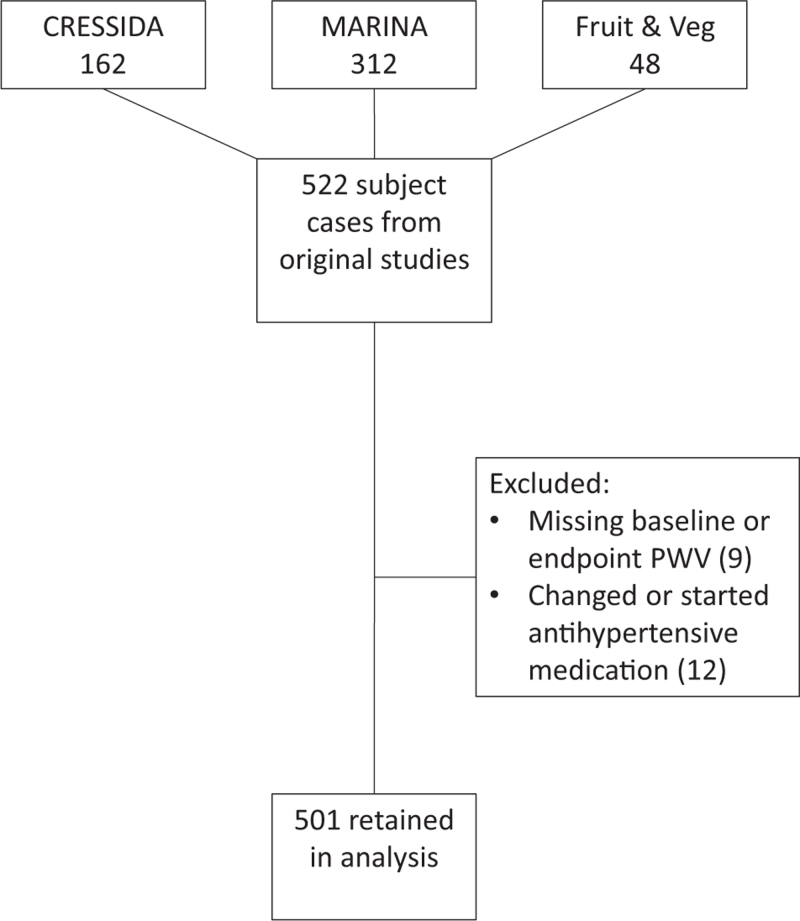
Consort diagram of the flow of individuals through the study. PWV, pulse wave velocity.

Data were eligible for inclusion and analysis if they fulfilled the following criteria: individuals must have had at least two ABPM and two PWV measurements, individual arms of each study did not show a significant change in BP measurements from baseline (statistical method detailed below) and no change in antihypertensive medications during the study. The second criterion ensured that any discrepancy between repeat measurements were secondary to measurement technique and physiological variability, rather than changes in an individual's true average BP (defined as a hypothetical estimate without measurement error and physiological variation [16]) resulting from dietary or other interventions. From the 522 available individual cases, 501 were retained in this analysis, as summarized in Fig. 1.

### Measurements

ABPM measurements were performed with the A&D TM-2430 device (ScanMed, Moreton-in-Marsh, Gloucestershire, UK) in all studies. CRESSIDA and the Fruit & Veg study took five measurements of ABPM, whilst MARINA recorded three. The first baseline measurement for CRESSIDA was followed by a second baseline measurement approximately 3 weeks later, and then further measurements at 4, 8 and 12 weeks after the second baseline measurement. In Fruit & Veg, the first two measurements were approximately 6 weeks apart, and then subsequent measurements were every 11 weeks. MARINA measured ABPM at baseline, then 6 months and 12 months later. A full schedule of events can be found in Table S1 (in Supplemental Digital Content). ABPM devices were programmed to take measurements every 30 min from 0700 to 2200 h and hourly between 2200 and 0700 h, but daytime and night-time periods were defined by each participant according to a sleep diary.

PWV was measured by applanation tonometry of the carotid and femoral arteries using the SphygmoCor device (Atcor Medical, Sydney, Australia) after at least 15 min of rest. Further details of study procedures and study outcomes can be found in the published articles [8–10].

### Nocturnal dipping

Nocturnal dip category was estimated for each ABPM session firstly according to a simple dichotomous outcome of dipper (night-time SBP fall ≥ 10% of daytime SBP) or nondipper (night-time SBP fall < 10% daytime SBP). Dipping status was then further defined according to the four classic dipping patterns (dipper: nocturnal SBP fall >10% of daytime SBP), reduced dipping (nocturnal BP fall 1–10% of daytime SBP), reverse dipping (increase in nocturnal SBP) and extreme dipping (nocturnal SBP fall >20% of daytime SBP) [17].

### Data analysis

To verify whether individual arms of studies were eligible to be included in this analysis, repeated-measures analysis of variance (ANOVA) was used to assess if there were significant differences in BP across each study timeframe. If ANOVA demonstrated significance less than 0.05, posthoc pairwise comparisons were performed using the Bonferroni method between the initial and last ABPM measurement. Study arms were eligible to be included if the overall ANOVA significance was more than 0.05, or if *P* value was less than 0.05 but with no significant difference between the first and last measurement (see Table S2, Supplemental Digital Content, which details the sequential BPs for each study arm, and the significance of any differences). As such, all arms of the studies were considered eligible to be included in this analysis.

Correlations were tested with Pearson's correlation coefficient unless stated otherwise. Comparison of individual characteristics at baseline and study endpoint were compared with paired *t*-tests. Logarithmic transformation was used to calculate the within-individual coefficient of variation (CoV) and corresponding 95% confidence interval (CI) as described by Bland and Altman [18,19] for daytime, night-time and 24-h BP and heart rate (HR), and PWV. CoV was compared between normotensive and hypertensive individuals. Normotension was defined as baseline daytime SBP (SBP_day_) less than 135 mmHg, whereas hypertension was defined as baseline SBP_day_ at least 135 mmHg.

Each individual had two to five measurements of SBP_day_, night-time SBP (SBP_night_), daytime DBP (DBP_day_) and night-time DBP (DBP_night_). The mean and standard deviation (SD) of these measurements was calculated for each individual. This intra-individual SD was used as an estimate of BPV for each individual. Multivariable linear regression models were used to analyse associations between patient characteristics and BPV, using an enter method.

Effect of regression to the mean (or adaptation to the ABPM device) was analysed using repeated-measures ANOVA in a subset of 199 individuals who had five ABPM measurements. Fleiss’ Kappa was calculated to determine agreement above chance in dipping categories.

Statistical tests were performed in SPSS version 25 (IBM, Chicago, USA), and significance defined as a *P* value less than 0.05. One author (L.K.) had access to all the data and takes responsibility for its integrity and the data analysis.

## RESULTS

### Baseline characteristics

Participant characteristics at baseline are summarized in Table 1 (*n* = 501). The cohort was predominantly female (61%), with mean (±SD) age 53.4 ± 8.0 years. Most individuals were of white ethnicity (80%). Mean clinic (seated) SBP and DBP were 124 ± 16 and 80 ± 10 mmHg, respectively. A small proportion of participants in the MARINA trial were on stable antihypertensive medication (4%). No individuals from CRESSIDA or Fruit & Veg were on antihypertensive therapy. Mean baseline PWV was 8.4 ± 1.6 m/s and mean baseline ambulatory SBP was 130 ± 13 mmHg during the day, 110 ± 14 mmHg at night and 125 ± 13 mmHg over 24 h. Mean baseline ambulatory DBP was 79 ± 8 mmHg for day, 65 ± 8 mmHg at night and 76 ± 7 mmHg over 24 h. Mean BMI at baseline was 26.0 ± 3.9 kg/m^2.^ There was no significant change in mean BMI over the duration of the studies (*P* = 0.938).

**TABLE 1:** Individual characteristics at baseline

	CRESSIDA (*n* = 159)	Fruit & Veg (*n* = 48)	MARINA (*n* = 294)	All (*n* = 501)
Age (years)	52.9 ± 8.0	45.2 ± 9.4	55.1 ± 6.6	53.4 ± 8.0
Female [*n* (%)]	96 (60)	25 (52)	184 (63)	305 (61)
Ethnicity				
White [*n* (%)]	133 (84)	29 (60)	239 (81)	401 (80)
Black [*n* (%)]	14 (9)	10 (21)	15 (5)	39 (8)
Asian [*n* (%)]	10 (6)	9 (19)	27 (9)	46 (9)
Other/mixed [*n* (%)]	2 (1)	0	13 (4)	15 (3)
BMI (kg/m^2^)	26.0 ± 3.8	28.4 ± 3.8	25.5 ± 3.9	26.0 ± 3.9
Antihypertensive use [*n* (%)]	0	0	11 (4)	11 (2)
PWV (m/s)	7.5 ± 1.2	7.9 ± 1.0	8.9 ± 1.7	8.4 ± 1.6
Clinic seated measurements:				
SBP (mmHg)	120 ± 16	129 ± 12	126 ± 16	124 ± 16
DBP (mmHg)	79 ± 10	87 ± 8	80 ± 10	80 ± 10
HR (bpm)	66 ± 9	73 ± 9	68 ± 9	68 ± 9
Ambulatory measurements:				
SBP_day_ (mmHg)	126 ± 13	139 ± 14	131 ± 13	130 ± 13
SBP_night_ (mmHg)	107 ± 14	116 ± 14	110 ± 13	110 ± 14
24-h SBP (mmHg)	122 ± 12	135 ± 13	126 ± 12	125 ± 13
DBP_day_ (mmHg)	77 ± 8	88 ± 7	79 ± 7	79 ± 8
DBP_night_ (mmHg)	64 ± 9	71 ± 8	65 ± 7	65 ± 8
24-h DBP (mmHg)	74 ± 7	85 ± 7	76 ± 7	76 ± 7
HR_day_ (bpm)	72 ± 9	76 ± 7	76 ± 8	75 ± 8
HR_night_ (bpm)	62 ± 9	65 ± 9	64 ± 8	63 ± 9
24-h HR (bpm)	70 ± 8	74 ± 7	73 ± 7	72 ± 8

Values represent means ± standard deviation, or number [percentage]. HR, heart rate; PWV, pulse wave velocity.

Baseline SBP_day_ and SBP_night_ were significantly correlated with age (*r* = 0.15, *P* = 0.001 and *r* = 0.14, *P* = 0.001 respectively), but DBP_day_ and DBP_night_ were not (*r* = 0.02, *P* = 0.70 and *r* = 0.85, *P* = 0.06). Baseline BMI was significantly correlated with all baseline pressure measurements (*r* = 0.26, *P* < 0.001 for SBP_day_, *r* = 0.29, *P* < 0.001 for SBP_night_, *r* = 0.16, *P* < 0.001 for DBP_day_ and *r* = 0.23, *P* < 0.001 for DBP_night_).

### Associations between blood pressure variability and mean pressures

**FIGURE 2:**
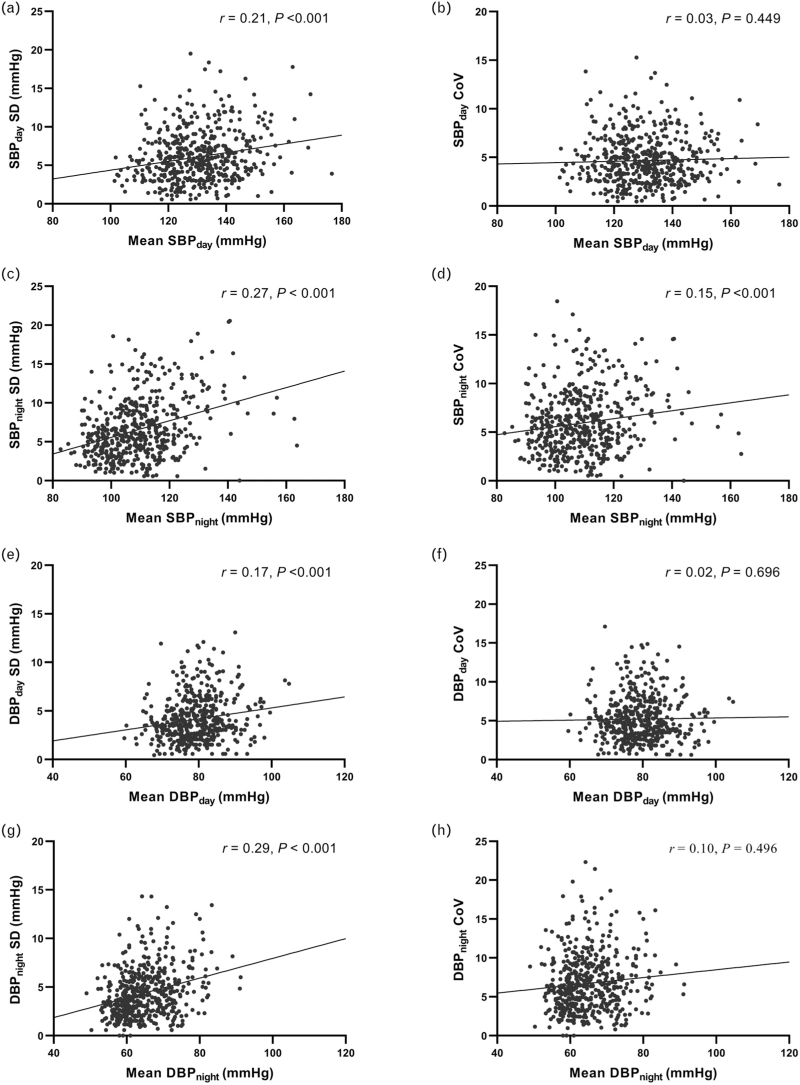
Associations between individual ambulatory mean pressures and SD or coefficient of variation. (a) Daytime systolic SD. (b) Daytime systolic CoV. (c) Night-time systolic SD. (d) Night-time systolic CoV. (e) Daytime diastolic SD. (f) Daytime diastolic CoV. (g) Night-time diastolic SD. (h) Night-time diastolic CoV. CoV, coefficient of variation; SD, standard deviation.

Significant associations were observed between mean ambulatory BP values and the variability of those measurements (Fig. 2). For SBP, both day and night measurements demonstrated a significant association between mean values and the SD of those measurements (SBP_day_*r* = 0.21, *P* < 0.001; SBP_night_*r* = 0.27, *P* < 0.001) (Fig. 2a and c). When the relationship was investigated using the ratio of variability and mean SBP (individual CoV), the strength of the relationship was no longer significant for day measurements (*r* = 0.03, *P* = 0.449) and reduced for night (*r* = 0.15, *P* = 0.001; Fig. 2b and d).

For ambulatory DBP measurements, a significant positive relationship was observed between mean values and the SD of those measurements (DBP_day_*r* = 0.17, *P* < 0.001 and DBP_night_*r* = 0.29, *P* < 0.001; Fig. 2e and g). Conducting the analyses with CoV removed the significant association for both DBP_day_ and DBP_night_ (*r* = 0.02, *P* = 0.696 and *r* = 0.10, *P* = 0.496, respectively; Fig. 2f and h).

### Within-individual coefficient of variation for repeated ambulatory blood pressure measurements

Measures of within-individual CoV for each study and for the entire cohort are summarized in Table 2. Qualitative analysis shows that measures of CoV for each BP measurement are similar for each study. In the entire cohort, the CoV for daytime measurements is significantly lower than that compared with night-time measurements: 5.4% (95% CI 5.2–5.6) for SBP_day_ compared with 7.0% (95% CI 6.7–7.3) for SBP_night_, and 6.1% (95% CI 5.9–6.4) for DBP_day_ compared with 8.4% (95% CI 8.0–8.7) for DBP_night_. CoV is significantly lower for 24-h ABPM measurements: 4.8% (95% CI 4.6–5.0) for SBP, and 5.3% (95% CI 5.1–5.5) for DBP.

**TABLE 2:** Measures of reproducibility in ambulatory blood pressure and pulse wave velocity

	CRESSIDA		Fruit & Veg		MARINA		ALL	
	*n*	CoV, % (95% CI)	*n*	CoV, % (95% CI)	*n*	CoV, % (95% CI)	*n*	CoV, % (95% CI)
All individuals	159		48		294		501	
SBP_day_		5.0 (4.8–5.4)		5.4 (4.9–6.0)		5.6 (5.3–5.9)		5.4 (5.2–5.6)
SBP_night_		6.7 (6.3–7.0)		7.5 (6.7–8.4)		7.1 (6.7–7.5)		7.0 (6.7–7.3)
24-h SBP		4.7 (4.4–4.9)		4.9 (4.4–5.5)		4.8 (4.5–5.1)		4.8 (4.6–5.0)
DBP_day_		5.1 (4.8–5.4)		6.3 (5.7–7.0)		6.6 (6.2–7.0)		6.1 (5.9–6.4)
DBP_night_		9.0 (8.5–9.5)		8.1 (7.2–8.9)		8.0 (7.5–8.5)		8.4 (8.0–8.7)
24-h DBP		4.8 (4.5–5.0)		5.7 (5.1–6.4)		5.5 (5.1–5.8)		5.3 (5.1–5.5)
HR_day_		6.0 (5.7–6.4)		6.5 (5.8–7.2)		6.4 (6.0–6.8)		6.3 (6.1–6.5)
HR_night_		7.2 (6.8–7.7)		7.8 (7.0–8.7)		8.3 (7.8–8.8)		7.9 (7.6–8.2)
24-h HR		5.8 (5.5–6.1)		6.0 (5.3–6.6)		5.9 (5.6–6.3)		5.9 (5.7–6.1)
PWV		7.6 (6.7–8.4)		10.4 (9.3–11.5)		12.1 (11.1–13.1)		10.7 (9.0–10.9)
Normotensive individuals	121		17		186		324	
SBP_day_		5.3 (4.9–5.6)		6.0 (5.0–7.0)		5.6 (5.2–6.0)		5.5 (5.2–5.8)
SBP_night_		6.7 (6.2–7.1)		7.2 (5.9–8.5)		7.0 (6.5–7.5)		6.9 (6.6–7.2)
24-h SBP		4.8 (4.5–5.1)		4.8 (3.9–5.6)		4.7 (4.3–5.1)		4.7 (4.5–5.0)
DBP_day_		5.3 (5.0–5.7)		7.7 (6.4–9.1)		6.4 (5.9–6.8)		6.1 (5.8–6.4)
DBP_night_		9.2 (8.6–9.8)		7.7 (6.3–9.1)		8.3 (7.7–8.9)		8.6 (8.2–9.0)
24-h DBP		5.0 (4.6–5.3)		6.5 (5.3–7.6)		5.3 (4.9–5.7)		5.3 (5.0–5.5)
HR_day_		6.1 (5.7–6.5)		6.5 (5.4–7.6)		6.3 (5.9–6.8)		6.3 (6.0–6.6)
HR_night_		7.4 (6.9–7.8)		7.0 (5.7–8.3)		8.7 (8.0–9.4)		8.1 (7.7–8.5)
24-h HR		6.0 (5.6–6.3)		5.4 (4.4–6.4)		5.8 (5.4–6.3)		5.9 (5.6–6.1)
PWV		7.1 (6.2–8.0)		11.0 (9.1–13.0)		11.7 (10.5–13.0)		10.2 (9.4–10.9)
Hypertensive individuals	38		31		108		177	
SBP_day_		4.4 (3.9–4.9)		5.1 (4.4–5.7)		5.6 (5.1–6.2)		5.3 (4.9–5.6)
SBP_night_		6.6 (5.8–7.4)		7.8 (6.7–8.8)		7.3 (6.5–8.0)		7.2 (6.7–7.7)
24-h SBP		4.1 (3.6–4.6)		5.0 (4.4–5.7)		5.1 (4.6–5.6)		4.9 (4.5–5.2)
DBP_day_		4.4 (3.9–4.9)		5.5 (4.8–6.2)		6.9 (6.2–7.6)		6.2 (5.8–6.6)
DBP_night_		8.2 (7.3–9.2)		8.3 (7.2–9.4)		7.6 (6.8–8.3)		7.8 (7.3–8.4)
24-h DBP		4.1 (3.6–4.6)		5.3 (4.6–6.0)		5.6 (5.1–6.2)		5.3 (4.9–5.6)
HR_day_		5.6 (5.0–6.3)		6.5 (5.7–7.3)		6.6 (5.9–7.2)		6.4 (6.0–6.8)
HR_night_		6.9 (6.1–7.7)		8.3 (7.2–9.3)		7.6 (6.8–8.4)		7.6 (7.1–8.1)
24-h HR		5.3 (4.7–5.9)		6.2 (5.4–7.1)		6.1 (5.5–6.7)		5.9 (5.6–6.3)
PWV		9.0 (6.9–11.1)		10.0 (8.7–11.4)		12.7 (10.9–14.5)		11.5 (10.5–12.6)

Normotension defined as baseline ambulatory SBP_day_ <135 mmHg. Hypertension defined as baseline ambulatory SBP_day_ ≥135 mmHg. CI, confidence interval; CoV, within-individual coefficient of variation; HR, heart rate; PWV, pulse wave velocity.

**FIGURE 3:**
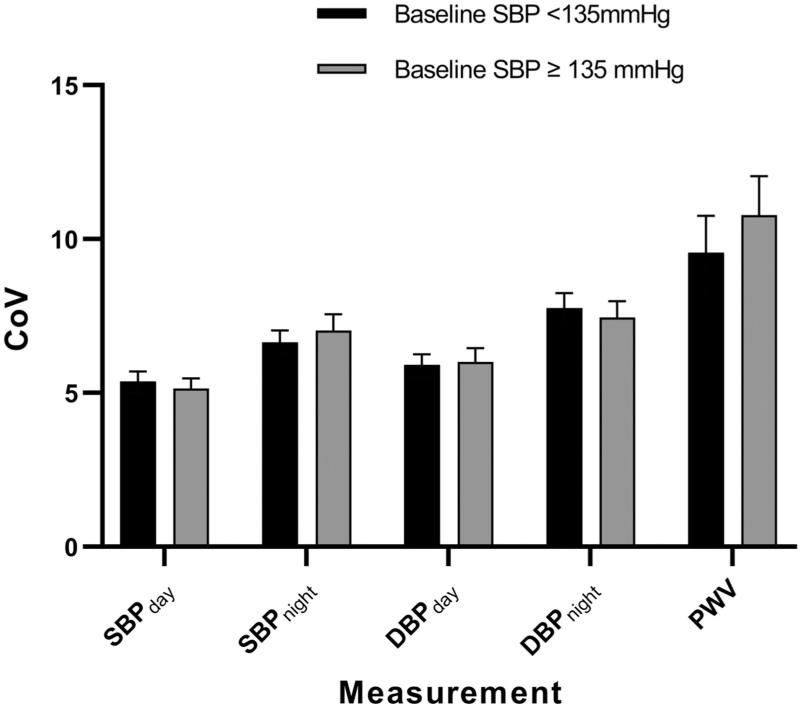
Comparison of within-individual coefficient of variation between individuals defined as normotensive at study baseline (SBP <135 mmHg) versus individuals defined as hypertensive at study baseline (SBP_day_ ≥135 mmHg). Error bars represent 95% confidence intervals. CoV, within-individual coefficient of variation; PWV, pulse wave velocity.

Reproducibility of ambulatory measurements was compared between individuals defined as normotensive on their baseline visit compared with those defined as hypertensive (Table 2 and Fig. 3). The mean baseline SBP for the normotensive group was 122 ± 8 mmHg compared with 144 ± 9 mmHg for the hypertensive group. When considering all normotensives versus all hypertensive individuals, there was no clear evidence of any difference in the reproducibility of SBP_day_, SBP_night_, DBP_day_ or DBP_night_. However, both the CRESSIDA and Fruit & Veg studies showed significantly less variability in hypertensive individuals than normotensive individuals for measurements of DBP_day_: 4.4% (95% CI 3.9–4.9) in hypertensive individuals compared with 5.3% (95% CI 5.0–5.7) in normotensive individuals in CRESSIDA, and 5.5% (95% CI 4.8–6.2) in hypertensive individuals compared with 7.7% (95% CI 6.4–9.1) in normotensive individuals in Fruit & Veg.

### Association of individual risk factors to individual blood pressure variability

Average estimates of individual BPV, as assessed by the SD, were as follows: SBP_day_ SD 6.1 ± 3.3 mmHg, SBP_night_ SD 6.6 ± 3.8 mmHg, DBP_day_ SD 4.1 ± 2.3 mmHg and DBP_night_ SD 4.4 ± 2.5 mmHg. BPV was not correlated with age for SBP_day_ SD, SBP_night_ SD, DBP_day_ SD, DBP_night_ SD (all *P* > 0.05). BMI at baseline was significantly correlated with SBP_day_ SD (*r* = 0.09, *P* = 0.04), SBP_night_ SD (*r* = 0.20, *P* < 0.001) and DBP_night_ SD (*r* = 0.19, *P* < 0.001), and had a borderline significant correlation with DBP_day_ SD (*r* = 0.09, *P* = 0.054).

Table 3 summarizes multivariable linear regression investigating the associations between BPV to individual demographics and mean BP. No significant associations were demonstrated for age, sex or PWV with SD for SBP_day_, SBP_night_, DBP_day_ or DBP_night_. SBP_day_ SD was independently associated with nonwhite ethnicity, use of antihypertensive medication and mean SBP_day_. SBP_night_ SD was independently associated with baseline BMI and mean SBP_night_. DBP_day_ SD was only associated with mean DBP_day_. DBP_night_ SD was independently associated with baseline BMI and mean DBP_night_. Further analyses were performed examining the effect of mean sleep duration on night-time variability, in a subset of 207 individuals in whom these data were available (the CRESSIDA and Fruit & Veg participants). Mean sleep duration was not independently associated with SBP_night_ SD (*P* = 0.482) or DBP_night_ SD (*P* = 0.160), as shown in Supplemental Digital Content, Table S3, which details the full linear regression models.

**TABLE 3:** Multivariable linear regression showing associations between variability of ambulatory blood pressures to mean blood pressure and demographic risk factors

	SBP_day_ SD		SBP_night_ SD		DBP_day_ SD		DBP_night_ SD	
	β	*P*	β	*P*	β	*P*	β	*P*
Age (years)	0.030	0.578	0.020	0.705	-0.062	0.256	0.088	0.094
Sex (male/female)	0.030	0.521	-0.042	0.349	0.040	0.397	0.014	0.763
Ethnicity (white/other)	**0.090**	**0.049**	0.023	0.608	0.029	0.542	0.009	0.838
BMI (kg/m^2^)	0.027	0.561	**0.097**	**0.033**	0.052	0.277	**0.136**	**0.003**
PWV (m/s)	-0.056	0.319	-0.092	0.088	-0.019	0.733	-0.106	0.055
Antihypertensives (yes/no)	**0.112**	**0.012**	-0.049	0.251	0.083	0.068	0.034	0.437
Mean SBP_day_ (mmHg)	**0.271**	**0.001**	–	–	0.023	0.709	–	–
Mean DBP_day_ (mmHg)	-0.079	0.279	–	–	**0.155**	**0.008**	–	–
Mean SBP_night_ (mmHg)	–	–	**0.414**	**<0.001**	–	–	0.063	0.456
Mean DBP_night_ (mmHg)	–	–	-0.093	0.245	–	–	**0.223**	**0.006**

B, standardized regression coefficient. PWV, pulse wave velocity; SD, standard deviation (measure of blood pressure variability). *P* < 0.05 highlighted in bold.

### Adaptation to the ambulatory blood pressure monitoring device

Adaptation to the ABPM device was tested in a subset of 199 individuals who had the full five measures of each BP. Repeated-measures ANOVA shows no evidence of adaptation to the device in terms of SBP_day_, SBP_night_ or DBP_day_ (all *P* > 0.05). However, DBP_night_ changed significantly over the course of sequential measurements, being at its lowest on the baseline visit, highest on second assessment, then decreasing sequentially (*P* = 0.001).

### Variability of arterial stiffness measurements

Baseline PWV was 8.4 ± 1.6 m/s, compared with 8.3 ± 1.6 m/s at study endpoint (*P* = 0.016). Mean PWV was positively correlated with mean ABPM values: SBP_day_ (*r* = 0.40, *P* < 0.001), SBP_night_ (*r* = 0.41, *P* < 0.001), DBP_day_ (*r* = 0.25, *P* < 0.001) and DBP_night_ (*r* = 0.30, *P* < 0.001).

Reproducibility of PWV measurements differed between studies (Table 2). There was a significantly lower CoV for PWV measured in the CRESSIDA study: 7.6% (95% CI 6.7–8.4) compared with the Fruit & Veg and MARINA studies: 10.4% (95% CI 9.3–11.5) and 12.1% (95% CI 11.1–13.1), respectively. In the total cohort, CoV of repeated PWV measurements was 10.7% (95% CI 9.0–10.9), with no statistical difference demonstrated between the PWV reproducibility between normotensive and hypertensive individuals: 10.2% (95% CI 9.4–10.9) versus 11.5% (95% CI 10.5–12.6).

The mean PWV and its SD were significantly and positively correlated (*r* = 0.28, *P* < 0.001). However, there were no significant correlations between mean PWV and BPV of SBP_day_, SBP_night_, DBP_day_ or DBP_night_.

### Variability of heart rate measurements

Baseline HR measured in clinic was 68 ± 9, compared with 75 ± 7 for the baseline HR measured by ABPM (*P* < 0.001). CoV of HR measurements was 6.3% (95% CI 6.1–6.5) during the day, 7.9% (95% CI 7.6–8.2) during the night and 5.9% (95% CI 5.7–6.1) over 24 h, as summarized in Table 2.

### Nocturnal dipping

Using the binary definition of dipper versus nondipper, 385 individuals (77%) were classed as normal dippers on their first ABPM measurement, with 100 (20%) classed as nondippers. Using the four standard categories of dipping, 243 individuals (49%) showed a normal dipping pattern, whilst 88 (18%) had reduced dipping, 142 (28%) showed extreme dipping and 12 (2%) showed reverse dipping on their baseline measurement.

The majority of normotensive and hypertensive individuals were classified as normal dippers on the binary classification at study baseline. Dippers accounted for 240 (74%) of normotensive individuals compared with 145 (82%) of hypertensive individuals. When considering dipping status over all available measurements, 1% of normotensive individuals were nondippers throughout, 45% were dippers throughout and 53% were changeable over their measurements, compared with 3% of hypertensives being nondippers throughout, 54% remaining a dipper throughout and 41% changing their status. There was a weak but significant agreement in dipping status for both normotensive and hypertensive individuals (*κ* = 0.132, *P* < 0.001 and *κ* = 0.187, *P* < 0.001, respectively) when analysed over five ABPM measurements (*n* = 194).

Using the four categories of dipping (reverse, reduced, normal and extreme), the majority of normotensive and hypertensive individuals were again classed as normal dippers (51 and 44%, respectively). Both groups also showed a tendency to change category over the course of their measurements. In the normotensive group, 271 (84%) changed their dipping category, and in the hypertensive group, 141 (80%) changed their dipping category. In individuals with the full five measurements, only 11% of normotensive individuals maintained their original dipping category (*κ* = 0.107, *P* < 0.001). Similarly, only 11% of hypertensive individuals maintained their original dipping category over five ABPM measurements (*κ* = 0.160, *P* < 0.001).

## DISCUSSION

To our knowledge, this is the largest study to examine reproducibility of serial ABPM measurements in a cohort of adults with minimal cardiovascular comorbidities. Reproducibility estimates are not dissimilar to those calculated by others. Our CoV estimates of 5.4 and 6.1% for daytime SBP and DBP, respectively, are close to the 5.5 and 4.9% calculated by Warren *et al.*[20] in a cohort of 163 individuals of similar age (although with a higher proportion of antihypertensive use) and lower than 7.4 and 6.3% calculated by Mansoor *et al.*[3] in their cohort of hypertensive patients (*n* = 25). Our night-time CoVs were slightly higher than those obtained by Mansoor *et al.*[3]: 7.0% compared with 6.3% for night-time SBP, and 8.4% compared with their 7.1% for night-time DBP [3]. Despite the large difference in baseline SBP_day_ between the normotensive and hypertensive group, we did not demonstrate any marked differences in ABPM measurement reproducibility in normotensive versus hypertensive individuals. By using CoV as a measure of reproducibility (rather than SD, which is correlated to mean BP), we show that in our cohort, ABPM measurements were no more variable in stable hypertensive individuals than in normotensive individuals when the mean BP was accounted for.

Variability of our night-time measurements generally exceeded that of daytime measurements, as also found by Bo *et al.*[21]. This could be attributable to inconsistency of nocturnal dipping patterns [22] or direct interruption of sleep due to the operation of the ABPM device. Poor sleep quality is associated with increased BPV [23] and with increased BP [24], but it is contentious whether ABPM devices impair sleep quality enough to produce a significant increase in nocturnal pressures [25,26]. We were not able to analyse the effect of sleep quality in this study, but sleep duration did not appear to have a significant effect on night-time variability. When we analysed patterns of nocturnal dipping, we found little agreement above chance in categorisation of dipping status. This trend persisted whether we used four categories of classification, or a simplified dichotomous classification, and with little difference seen between normotensive and hypertensive individual groups. Although abnormal nocturnal dipping has been shown to be associated with adverse cardiovascular outcomes [27], its poor reproducibility shown by ourselves and others [21,22,28] may limit its use for stratifying risk. As many studies on nocturnal dip variability examine only two measurements, further large studies are needed to examine reproducibility of nocturnal dip over multiple measurements with an emphasis on determining subgroups particularly prone to high variation.

We have shown that BPV, an important predictor of cardiovascular risk, is positively associated with mean BP but were unable to demonstrate any significant associations with age, sex or concurrent arterial stiffness when the mean BP was accounted for. Arterial stiffening may be a long-term consequence rather than a cause of BPV [15], hence the lack of association seen in cross-sectional regression. Increased BMI was associated with a higher baseline BP and increased variability of night-time measurements, but not daytime pressures, which may reflect findings by others that higher BMI is associated with increased BPV, and disruption of normal nocturnal dipping patterns [29,30]. White participants appeared to have less variability in their SBP_day_ measurements compared with nonwhite ethnicity individuals, in agreement with other studies showing that African–Americans have higher BPV than white individuals, as well as higher mean ambulatory pressures [31], for which several physical and socioeconomic reasons have been suggested [32].

A secondary aim of this study was to examine if the variability of arterial stiffness, as measured by PWV, was superior to that of ABPM. Overall, PWV was found to have a CoV of 10.7% for the whole cohort, which is similar to that found by others in short-term studies [33], but higher than the CoV for the BP measures, which ranged from 5.4 to 8.4%. Coupled with the fact that PWV requires specialist equipment and user training, this suggests that, unless it is more strongly related to risk of clinical outcomes, it is not preferable as a surrogate measurement for long-term BP monitoring. PWV measurements appeared more variable in hypertensive compared with normotensive individuals, but this is to be expected given that mean and SD values of PWV were correlated, and PWV is itself highly correlated with concurrent BP.

Use of ABPM is becoming more widespread, as current guidelines recommend its use to confirm a new diagnosis of hypertension [34,35]. However, for long-term monitoring of BP, NICE still advises use of clinic BP measurement, with ABPM suggested as a confirmatory tool for individuals who could have white-coat or masked hypertension [35]. Reproducibility of repeated ABPM has been studied, but often in small cohorts and a wide range of reproducibility indices used across the literature. Our cohort was composed of individuals with minimal cardiovascular morbidities and who did not require initiation or alteration of antihypertensive medication during the study period. In such individuals, it could be hypothesized that variability of BP measurements should be minimal. However, we have shown that the within-individual variability of ABPM measurements is still large when considered in clinical context. A borderline hypertensive clinic individual may be given ABPM to confirm or refute the presence of true hypertension. If their true daytime SBP was 140 mmHg, however, a CoV of 5.4% for SBP_day_ by ABPM implies that 95% of readings will normally occur within a range of 125–155 mmHg, making diagnosis uncertain. Similarly, for a true daytime diastolic pressure of 90 mmHg, 95% of measurements would occur within a range of 78–102 mmHg (based on a CoV of 6.1%). Night-time estimates may be subject to even greater variability, as we have noted that the CoV of night-time measurements is significantly higher than those found during the day. Currently, NICE only recommends use of daytime ABPM to guide diagnosis [35], but future work could explore the use of night-time and 24-h BP to guide antihypertensive therapy, as nocturnal BP is correlated with cardiovascular outcomes [36,37], and variability of 24-h BP is less than daytime BP, as shown in this work and others [4,38–40].

Clinicians should note that SD of measurements is proportional to mean pressure and precise assessment of BP in a hypertensive individual may therefore be subject to additional complexity. An additional consideration in the use of single ABPM measurements to guide treatment is the possibility of an adaptive response to the device, whereby the first use elicits an additional pressor response with subsequent values showing regression to the mean. Although we were unable to show evidence of adaptation in terms of SBP_day_, SBP_night_ or DBP_day_, we did note some changes in DBP_night_ over the course of sequential measurements and nocturnal ABPs have been shown to be susceptible to adaptation as well as daytime measurements [38,41].

Our recent work using Monte Carlo simulations of BP treatments showed that measurement error is the main cause for misclassification of BP target when undertaking stepwise titration of antihypertensive therapy [16,42]_._ Readings of low error are likely to improve BP control, a conclusion supported by general consensus [43,44]. It is interesting to note that the measurement margins calculated here are in excess of the likely response to antihypertensive monotherapy (∼ 9.1 mmHg SBP, ∼5.5 mmHg DBP), and may even exceed that expected for dual therapy in some instances [45], highlighting the limitations of single ABPM measurements.

### Limitations

The present study is subject to several important limitations. Firstly, this study uses retrospective data from interventional studies, which were designed to detect differences from baseline in ABPM and PWV, rather than assess variability within a stable population over time. Furthermore, the three studies differ in design and so the extent to which their data are directly comparable must be considered. The analyses presented here were designed to mitigate against these potential issues. Firstly, each study arm was only included if there was no significant change in measurements from baseline. This is a different approach to that used within each study, which generally compared intervention. In the CRESSIDA study this showed a significant 4.2 mmHg reduction in ambulatory daytime SBP in the intervention compared to control group. Our approach was defined *a priori* and was designed to maximise the data available, albeit with a recognition that various interventions may have an unknown impact on measures of interest. For example, we note that subsequent analysis from the MARINA study has identified that genotype may have dictated an individual's response to the fish oils given [46]. However, even with a potential postintervention increase up to 5 mmHg on endpoint SBP, our CoV estimates for SBP would not be significantly altered (calculation not shown). Secondly, we used IPD rather than summary data to provide more reliable results [47]. Thirdly, the limited number of repeat measurements for each participant may have inflated true values for individual variability but does approximate better to clinical practice than a high number of repeated ABPMs. The consistency of results between the three different studies provides some reassurance for our approach and the comparability of the datasets.

In conclusion, this study highlights that although ABPM is the gold standard for BP measurement and monitoring, variability between measurements may result in misclassification and incorrect treatment decisions. Within our analysis population, PWV measurement was not a more reproducible technique than ABPM when assessed as a CoV. Repeated ABPM may be necessary to refine antihypertensive therapy.

## ACKNOWLEDGEMENTS

This work was supported by the Medical Research Council and British Heart Foundation [MR/M016560/1]. The authors acknowledge financial support from the Department of Health via the National Institute for Health Research (NIHR) comprehensive Biomedical Research Centre and Clinical Research Facilities awards to Guy's and St Thomas’ NHS Foundation Trust in partnership with King's College London and King's College Hospital NHS Foundation Trust.

This work has not been published in its current form or a substantially similar form, has not been accepted for publication elsewhere and is not under consideration by another publication.

Data supporting this article are not openly available due to ethical restrictions. A descriptive record can be found in the Kings College London research data repository at https://doi.org/10.18742/20348892. Data may be shared on reasonable request by application to Professor Tom Sanders, tom.sanders@kcl.ac.uk.

### Conflicts of interest

The authors declare no conflicts of interest.

## Supplementary Material

Supplemental Digital Content
