# Supplementary material for: Reproducibility of sequential ambulatory blood pressure and pulse wave velocity measurements in normotensive and hypertensive individuals
Source: J Hypertens. 2022 Sep 27;40(12):2528–37. doi: 10.1097/HJH.0000000000003290 (PMC9640295; doi:10.1097/HJH.0000000000003290)
Supplement: Supplemental Digital Content [file jhype-40-2528-s001.pdf]

Table S1: Schedule of assessments for included studies

	Summary of ABPM and PWV assessments				
	**1**	**2**	**3**	**4**	**5**
**Parallel design, randomised controlled trials**					
MARINA	Baseline ABPM and PWV	Midpoint ABPM at 6 months	End-of-study ABPM and PWV at 12 months	-	-
CRESSIDA	First baseline ABPM	Second baseline 2 ABPM and PWV 23 ± 16 days after first baseline	Midpoint ABPM 4-6 weeks after second baseline	Midpoint ABPM 8 weeks after second baseline	End-of-study ABPM and PWV 12 weeks after second baseline
**Randomised, placebo-controlled crossover**					
Fruit & Veg	Baseline ABPM and PWV	Intervention A: ABPM and PWV 6 weeks after baseline	Intervention B: ABPM and PWV 17 weeks after baseline	Intervention C: ABPM and PWV 28 weeks after baseline	Intervention D: ABPM and PWV 39 weeks after baseline

ABPM: ambulatory blood pressure monitoring, PWV: pulse wave velocity

Table S2: Mean values of sequential ABPM measurements for each study arm.

	Sequential ABPM Measurements					
	**1**	**2**	**3**	**4**	**5**	***P***
**CRESSIDA**						
**Control (n = 81)**						
SBP_{day}	127.2 ± 12.4	126.8 ± 12.8	126.8 ± 12.8	128.0 ± 12.8	128.8 ± 13.2	0.223
SBP_{night}	109.8 ± 14.5	109.7 ± 13.4	109.3 ± 13.4	109.7 ± 12.9	109.3 ± 11.4	0.979
DBP_{day}	77.4 ± 7.9	77.0 ± 7.8	77.4 ± 8.2	77.5 ± 8.2	77.8 ± 8.2	0.695
DBP_{night}	64.6 ± 8.9	68.0 ± 9.1	67.5 ± 10.4	66.9 ± 9.6	66.6 ± 9.5	**0.015**[*]
**Dietary Guidance (n= 78)**						
SBP_{day}	124.3 ± 13.5	125.3 ± 14.4	124.1 ± 13.1	124.1 ± 14.0	122.7 ± 13.7	0.185
SBP_{night}	105.2 ± 12.9	105.9 ± 13.7	104.2 ± 11.9	104.5 ± 12.1	104.0 ± 12.8	0.386
DBP_{day}	75.9 ± 7.6	76.5 ± 7.9	76.1 ± 7.5	76.0 ± 7.4	74.8 ± 7.3	0.102
DBP_{night}	62.9 ± 8.4	65.3 ± 9.5	64.4 ± 9.0	64.7 ± 8.6	64.1 ± 9.7	0.105
**MARINA**						
**Placebo (n= 66)**						
SBP_{day}	133.6 ± 11.9	135.9 ± 15.0	131.8 ± 13.8	-	-	**0.021**[†]
SBP_{night}	111.0 ± 12.7	111.3 ± 13.3	110.5 ± 14.6	-	-	0.855
DBP_{day}	81.2 ± 6.9	81.7 ± 8.6	79.3 ± 7.3	-	-	0.031
DBP_{night}	66.1 ± 7.7	65.2 ± 7.0	65.2 ± 9.1	-	-	0.584
**0.45g/day (n=76)**						
SBP_{day}	129.6 ± 12.8	128.1 ± 11.8	130.0 ± 11.8	-	-	0.204
SBP_{night}	107.8 ± 11.1	107.5 ± 12.9	108.3 ± 12.4	-	-	0.803
DBP_{day}	77.8 ± 7.0	77.8 ± 6.9	78.8 ± 7.4	-	-	0.374
DBP_{night}	63.9 ± 7.0	62.4 ± 7.0	64.8 ± 7.9	-	-	**0.015**[‡]
**0.9g/day (n = 74)**						

SBP_{day}	132.3 ± 12.3	132.5 ± 11.8	132.1 ± 13.5	-	-	0.958
SBP_{night}	112.3 ± 15.5	110.6 ± 15.0	111.6 ± 13.5	-	-	0.416
DBP_{day}	80.2 ± 7.8	80.6 ± 6.8	79.9 ± 6.9	-	-	0.697
DBP_{night}	66.3 ± 7.6	65.6 ± 8.0	66.1 ± 6.7	-	-	0.678
**1.8g/day (n = 77)**						
SBP_{day}	129.2 ± 13.0	130.0 ± 12.6	128.6 ± 11.9	-	-	0.438
SBP_{night}	108.0 ± 13.7	106.2 ± 14.0	105.8 ± 12.4	-	-	0.165
DBP_{day}	79.0 ± 6.8	79.5 ± 6.5	79.8 ± 7.5	-	-	0.575
DBP_{night}	64.5 ± 7.5	62.3 ± 7.2	62.7 ± 7.2	-	-	**0.016**‖
**Fruit & Veg (n =48)**						
SBP_{day}	138.6 ± 14.0	139.5 ± 12.7	139.4 ± 13.1	136.7 ± 12.5	137.8 ± 14.1	0.287
SBP_{night}	116.7 ± 14.1	119.1 ± 16.7	119.1 ± 14.5	115.8 ± 14.3	118.4 ± 15.5	0.295
DBP_{day}	87.6 ± 7.4	87.9 ± 8.2	87.3 ± 7.9	86.5 ± 7.4	86.7 ± 9.6	0.604
DBP_{night}	70.6 ± 8.0	69.9 ± 7.8	72.3 ± 8.3	72.2 ± 10.4	71.3 ± 9.5	0.274

* Post-hoc pairwise comparison with Bonferroni method shows no significant difference in ABPM 1 vs ABPM 5 ($P = 0.942$).

† Post-hoc pairwise comparison with Bonferroni method shows no significant difference in ABPM 1 vs ABPM 3 ($P = 0.360$)

‡ Post-hoc pairwise comparison with Bonferroni method shows no significant difference in ABPM 1 vs ABPM 3 ($P = 0.790$)

|| Post-hoc pairwise comparison with Bonferroni method shows no significant difference in ABPM 1 vs ABPM 3 ($P = 0.105$)

Values represent means ± standard deviation. $P < 0.05$ highlighted in bold.

SBP: systolic blood pressure, DBP: diastolic blood pressure, ABPM: ambulatory blood pressure monitoring

Table S3: Multivariable linear regression showing associations between variability of ambulatory blood pressures to mean blood pressure and demographic risk factors, including sleep duration.

	SBP_{night} SD		DBP_{night} SD	
	**β**	***P***	**β**	***P***
Age (years)	0.126	0.096	0.137	0.080
Sex (male/female)	0.019	0.777	0.045	0.520
Ethnicity (white/other)	0.102	0.135	0.068	0.336
BMI (kg/m^2)	0.180	0.010	0.228	0.002
PWV (m/s)	-0.121	0.127	-0.091	0.266
Mean SBP_{night} (mmHg)	0.489	<0.001	0.643	0.521
Mean DBP_{night} (mmHg)	-0.068	0.601	0.257	0.056
Mean sleep duration (minutes)	-0.045	0.482	-0.094	0.160

β: standardised regression coefficient. SD: standard deviation (measure of blood pressure variability), BMI: body mass index, PWV: pulse wave velocity, SBP: systolic blood pressure, DBP: diastolic blood pressure. *P* <0.05 highlighted in bold.
